# LONGITUDINAL ASSOCIATIONS OF HEARING PROBLEM AND DEMENTIA IN COMMUNITY-DWELLING OLDER ADULTS IN THE UNITED STATES

**DOI:** 10.1093/geroni/igae098.3865

**Published:** 2024-12-31

**Authors:** Jun Chu, Christine Mair, Michel Boudreaux

**Affiliations:** University of Maryland, Baltimore County Baltimore, Maryland, United States; University of Maryland, Baltimore County Baltimore, Maryland, United States; University of Maryland College Park, College Park, Maryland, United States

## Abstract

There is evidence linking hearing problem to greater risk of dementia among older adults. However, no study has assessed such association longitudinally using nationally representative survey data. In addition, studies examining racial/ethnic differences in the association are limited. Using data from 2011-2022 National Health and Aging Trends Study and cox proportional hazards model, we examined the longitudinal association between having hearing trouble and dementia among older adults, and whether this association varied by race the ethnicity. Adjusting for demographics characteristics, number of chronic conditions, hearing aid status and social isolation status, we found that having hearing trouble was significant associated with a 2.29 (Hazard ratio=2.29, p< 0.001) higher risk of developing dementia over 11 years. After interacting race/ethnicity and hearing problem in a separate model, we did not find any statistically significant difference in the association by race and ethnicity. The findings suggest that older adults with hearing problem may be at a greater risk of developing dementia. Future studies should assess the effects of policies that promotes hearing tests and hearing aid usage among older adults on dementia delay and prevention.

